# Cerebral Hemodynamic Changes of Mild Traumatic Brain Injury at the Acute Stage

**DOI:** 10.1371/journal.pone.0118061

**Published:** 2015-02-06

**Authors:** Hardik Doshi, Natalie Wiseman, Jun Liu, Wentao Wang, Robert D. Welch, Brian J. O’Neil, Conor Zuk, Xiao Wang, Valerie Mika, Jerzy P. Szaflarski, E. Mark Haacke, Zhifeng Kou

**Affiliations:** 1 Department of Biomedical Engineering, Wayne State University, Detroit, Michigan, United States of America; 2 Department of Psychiatry and Behavioral Neurosciences Translational Neuroscience Program, Wayne State University School of Medicine, Detroit, Michigan, United States of America; 3 Department of Radiology, Second Xiangya Hospital, School of Public Health, Central South University, Changsha, Hunan Province, China; 4 College of Computer Science, South-Central University for Nationalities, Wuhan, China; 5 Department of Emergency Medicine, Wayne State University School of Medicine, Detroit, Michigan, United States of America; 6 Department of Radiology, Wayne State University School of Medicine, Detroit, Michigan, United States of America; 7 Zhengzhou University First Affiliated Hospital, Zhengzhou, Henan Province, China; 8 Department of Neurology, University of Alabama at Birmingham, Birmingham, Alabama, United States of America; University Of Cambridge, UNITED KINGDOM

## Abstract

Mild traumatic brain injury (mTBI) is a significant public health care burden in the United States. However, we lack a detailed understanding of the pathophysiology following mTBI and its relation to symptoms and recovery. With advanced magnetic resonance imaging (MRI), we can investigate brain perfusion and oxygenation in regions known to be implicated in symptoms, including cortical gray matter and subcortical structures. In this study, we assessed 14 mTBI patients and 18 controls with susceptibility weighted imaging and mapping (SWIM) for blood oxygenation quantification. In addition to SWIM, 7 patients and 12 controls had cerebral perfusion measured with arterial spin labeling (ASL). We found increases in regional cerebral blood flow (CBF) in the left striatum, and in frontal and occipital lobes in patients as compared to controls (p = 0.01, 0.03, 0.03 respectively). We also found decreases in venous susceptibility, indicating increases in venous oxygenation, in the left thalamostriate vein and right basal vein of Rosenthal (p = 0.04 in both). mTBI patients had significantly lower delayed recall scores on the standardized assessment of concussion, but neither susceptibility nor CBF measures were found to correlate with symptoms as assessed by neuropsychological testing. The increased CBF combined with increased venous oxygenation suggests an increase in cerebral blood flow that exceeds the oxygen demand of the tissue, in contrast to the regional hypoxia seen in more severe TBI. This may represent a neuroprotective response following mTBI, which warrants further investigation.

## Introduction

Over 1.7 million Americans suffer a traumatic brain injury (TBI) each year, most of which are classified as mild TBI (mTBI), also called concussion [1]. Despite its name, mTBI is not inconsequential [2–5]. The prolonged post-concussive symptoms significantly affect the quality of life of both patients and their families. However, the majority of patients with mTBI lack structural imaging correlates [6] thus making the clinical detection and outcome prediction a challenge. Following the initial traumatic insult, a series of vascular responses and perfusion changes is set in motion. Insults to the cerebral vasculature, such as microbleeds, local perfusion reduction, or oxygen metabolism changes are known to cause devastating secondary complications after moderate to severe TBI [7,8]. Further, perfusion studies using either arterial spin labeling (ASL) [9] or contrast-enhanced perfusion weighted imaging (PWI) [10] have demonstrated reduced cerebral blood flow in the chronic stages of mTBI.

Cerebral perfusion and oxygenation are of particular interest as markers of brain function, as neurons rely nearly completely on aerobic metabolism for their energy production. However, decreases in perfusion or venous oxygenation in the brain could indicate either a potentially at-risk area with unmet energy demands or a change in energy demands (a result of a change in function), which represent two entirely different injury responses with different implications for outcome. These hypoperfused areas can be identified by decreased CBF and oxygenation [11]; in patients with moderate to severe TBI ischemic volume has been shown to correlate with negative outcomes [12]. Additionally, early impairment of CBF in patients with severe injury correlates with poor brain tissue oxygenation [13]. Because of this, Zwienenberg and colleagues [14] have suggested optimizing cerebral perfusion and blood flow in the treatment of head injury patients. In mTBI, however, there is a lack of published data on the relationship between CBF and prognosis, as well as a lack of methods to distinguish primary alterations in CBF from primary alterations in brain metabolic demand. In particular, there is a lack of investigation of CBF changes in patients with mTBI in the acute stage (within 48 hours) of injury.

Further, there is paucity of data on blood oxygenation investigation in TBI patients and the extent of neurovascular compromise in mTBI, particularly of the early hemodynamic response in the acute stage, is still largely unknown. Due to the decoupling of cerebral blood supply and metabolic demand, measurement of CBF alone may not reflect the metabolic status of brain tissue. Ischemic/hypoxic brain tissue will have increased oxygen extraction, which results in decreased blood oxygenation in draining veins. Together with CBF, a measurement of venous blood oxygenation may help to paint a comprehensive picture of the hemodynamics of the brain tissue.

There have been attempts to extract oxygen saturation using T2, T2* or T2' approaches [15–31]. MR phase-based methods [32–37] have also been used on individual veins in an attempt to extract venous susceptibility and oxygen saturation. Another method is quantitative susceptibility mapping (QSM) which provides measures of oxygen extraction fraction [38,39]. Recently, Haacke et al have developed a method called susceptibility weighted imaging and mapping (SWIM), which is a form of QSM, for quantification of venous blood oxygenation [40]. Unlike other phase-based methods, the application of a regularized inverse filter to the frequency domain of the phase images results in susceptibility maps that are unaffected by the orientation of the blood vessels [40], providing a strong advantage over other phase-based measurements of susceptibility.

By pairing ASL with SWIM, it is possible to assess both perfusion and oxygenation of the injured brain. Thus, in the present study, we have assessed both CBF and venous oxygenation of several brain regions in which abnormalities are known to correlate with symptoms, including frontal, parietal, and temporal gray matter [41], cingulate cortex [42], and the basal ganglia [41], as well as occipital gray matter, thalamus, and striatum. We sampled the susceptibility of major veins that drain these and other areas including septal, thalamostriate, internal cerebral, and the basal veins of Rosenthal. We hypothesized that, after concussion, patients with mTBI may demonstrate reduced cerebral blood flow and decreased venous blood oxygenation in the acute stage. Additionally, we administered a short neurocognitive assessment, the standardized assessment of concussion (SAC) [43], to examine the correlation between the cognitive deficits and the imaging abnormalities.

## Methods

### Subject selection

This study was approved by both the Human Investigation Committee of Wayne State University and the Institutional Review Board of Detroit Medical Center. Written informed consent was obtained from each subject before enrollment.

A cohort of patients was prospectively recruited from the Detroit Receiving Hospital Emergency Department (ED), which is part of the Level-1 trauma center in the Detroit Medical Center. Patient eligibility was based on the mTBI definition by the American Congress of Rehabilitation Medicine [44] with the following inclusion criteria: Patients aged 18 or older with the initial Glasgow Coma Scale (GCS) score of 13–15 in ED with any period of loss of consciousness less than 30 minutes or any post traumatic amnesia less than 24 hours, or recorded change of mental status (confused, disoriented or dazed). All patients required a CT scan as part of their clinical evaluation. All patients were be able to communicate in English. The exclusion criteria included patients under the age of 18 years, pregnancy, medically documented history of previous brain injury, neurological disorders or psychoactive medications, history of substance abuse, CT indication of any metal in the brain and body, known contraindication to MRI (such as a pacemaker or other non-MR compatible implanted device) as identified by safety screening, or presentation without a clear history of trauma as their primary event (e.g., seizure, epilepsy, etc.). The patients' records were retrospectively screened as well to exclude any patient who does not fit our inclusion criteria.

A total of 14 patients with mTBI were recruited from the ED and 18 healthy controls were recruited via local advertising and from patients’ friends and relatives, as part of a study sponsored by the Department of Defense. Among them, SWI and structural images were acquired for all subjects and ASL data were obtained for 7 patients and 12 controls (Table 1), as the scanning protocol was amended to include the ASL sequence shortly after the initial study began. ASL data from two patients were excluded from the final analysis due to excessive motion during the scan. For mTBI patients, neurocognitive status was measured using the SAC questionnaire, which includes measures of attention, orientation and memory status.

**Table 1:** ASL-assessed patients’ and controls’ demographic data and cause of injury.

Case ID	Age (Years)	Sex	Race	Delay to Scan	ER GCS	Injury Mech.	ASL	SWI	Structural findings
Patients									
001	27	F	Caucasian	41 hr		MVA	X	X	
2	29	M	Caucasian	10 d	15	MVA		X	
002	24	M	Caucasian	46 hr		Fall		X	
4	20	M	Caucasian	27 hr	15	Fall		X	Moderate bleed, scalp edema
6	21	M	Black	4 d		MVA		X	
7	25	M	Black	17 hr	15	Assault	X	X	Nonsp hyperint, scalp edema
9	56	M	Indian	26 hr	15	MVA		X	Nonsp hyperint, 2 small bleeds
11	35	M	Black	36 hr		Fall		X	
14	30	M	Caucasian	7 d	15	Fall	X	X	
15	36	F	Black	9 hr	15	MVA	X	X	Arachnoid cyst
16	19	M	Black	3 hr	15	MVA	X	X	Pericallosal lipoma
17	23	M	Black	9 hr	15	MVA	X	X	
18	21	F	Caucasian	2 d	15	MVA		X	
19	30	F	Asian	8 hr	15	SBV	X	X	
Mean	27.14			55.29					
SD	5.52			68.82					
Median	26			31.5 hr					
Range	19–56			3 hr—10 d					
Controls									
1	24	F	Asian				X	X	
2	23	M	Indian				X		
3	45	M	Caucasian					X	Nonsp hyperint
5	22	M	Caucasian					X	Capillary telangiectasia
6	27	M	Asian				X	X	
7	23	F	Asian				X	X	
8	22	F	Asian				X	X	
9	65	F	Asian					X	Pineal gland cyst, Nonsp hyperint
36	52	F	Caucasian				X	X	Nonsp hyperint
37	44	M	Caucasian				X	X	Calcification of falx
38	41	M	Caucasian				X	X	
39	28	F	Caucasian					X	
40	27	F	Arabic				X	X	
41	29	M	Caucasian				X	X	
42	33	M	Caucasian				X	X	
43	66	F	Caucasian					X	Nonsp hyperint
44	38	M	Hispanic					X	Nonsp hyperint
45	28	M	Caucasian					X	
46	21	M	Black				X	X	
Mean	30.08								
SD	10.24								
Median	28								
Range	21–66								

hr = hours, d = days, nonsp hyperint = nonspecific hyperintensity, SBV = pedestrian struck by vehicle.

Data on patients’ blood alcohol levels, drug use, ED-administered medications, and blood pressure (BP) were collected from clinical records for inclusion in the final analyses as possible confounders.

### Imaging

All imaging was performed on a Siemens 3T VERIO magnet with a 32 channel head coil. The imaging protocol included a standard 3D T1 MPRAGE, T2 Fluid-attenuated inversion recovery (FLAIR), diffusion tensor imaging (DTI), SWI, ASL, and resting state functional MRI. This paper focuses on the hemodynamic analysis of brain injury using SWI and ASL data. All structural MRI images, including SWI images, were reviewed by two board-certified neuroradiologists to identify other conditions that could confound the results of the study. The neuroradiogists were blinded to the medical history and clinical condition of the subjects to avoid bias.

Arterial spin labeling. A pulsed ASL sequence was used, with a repetition time (TR) of 2830 milliseconds and echo time (TE) of 11 milliseconds, flip angle of 90 degrees, field of view of 384x384, and in-plane resolution of 4x4x4. After acquisition, the perfusion data were processed automatically by the Siemens online software to produce motion corrected relative CBF (rCBF) images for off-line post-processing.

SWI. The SWI sequence was a T2* Gradient Recalled Echo (GRE) based sequence with long TE and 3D flow compensation. The SWI data were collected with a TR/TE of 39/20 milliseconds, flip angle of 20 degrees, field of view of 256x256, and in-plane resolution of 0.5x1x2. It was later interpolated to 0.5x0.5x2 in-plane resolution.

### Image processing

**Fig 1:**
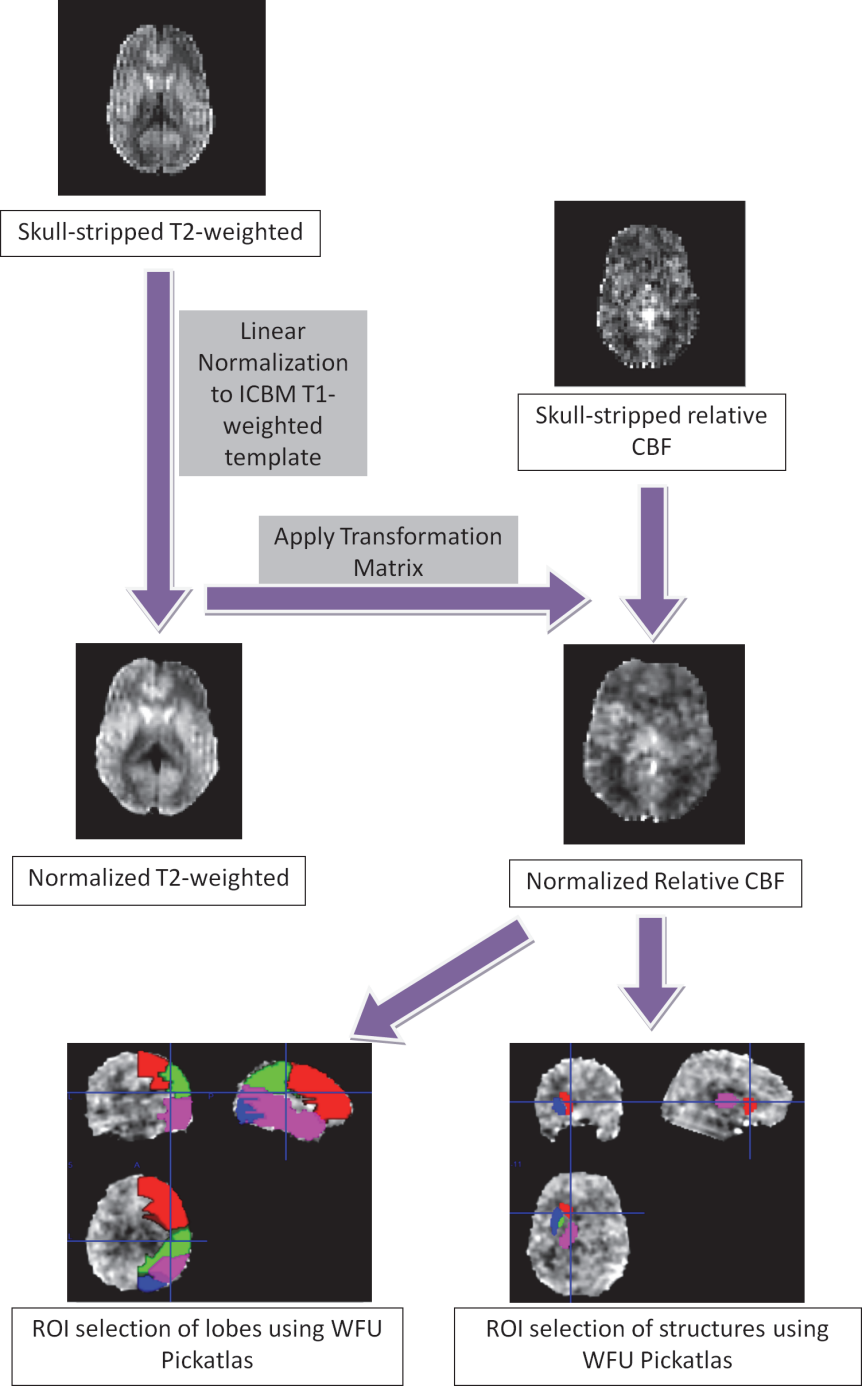
Diagram of the ASL analysis workflow. T2 images were skull-stripped and normalized to the ICBM T1-weighted template. A transformation matrix was applied to skull-stripped rCBF images with SPM8, to bring them into the same template. ROIs were selected automatically in the lobes and in deep brain structures using the Wake Forest University (WFU) PickAtlas.

ASL processing and analysis. A semi-automated process was used to select the regions of interest (ROIs) to avoid errors in manual selection of the ROIs (Fig. 1). Untagged T2-weighted images in ASL dataset and rCBF images were skull-stripped. T2 images were normalized to the T1-weighted International Consortium for Brain Mapping (ICBM) template, and a transformation matrix was applied to the relative rCBF images to bring them into the same standard space. A MATLAB-based Statistical Parametric Mapping 8 (SPM8) package was used for normalization [45–47].

After bringing all the images into the ICBM standard space, ROIs were automatically selected using the Wake Forest University PickAtlas (WFU pickatlas) [48]. Using these predefined ROIs in the ICBM space, regional rCBF values from the striatum, caudate nucleus, thalamus, globus pallidus, putamen, and frontal, occipital, parietal and temporal lobes of each subject were recorded.

### SWIM processing and analysis

Based on the previously described procedure [40,49], the following steps were taken for SWIM processing: a) skull stripping to remove the artifacts caused by skull and brain tissue interface by using the software package MRIcro (MRIcro, Version 1.40) with the Brain Extraction Tool (BET) [50]; b) phase unwrapping to extract SWI phase signal; c) background field removal to correct the background field inhomogeneity according to the method by Pandian et al. in 2008 [51]; d) inverse filtering to extract susceptibility signal; and e) iterative reconstruction to remove remnant ringing of potential microhemorrahges. This resulted in a high resolution susceptibility map of the venous structures of the brain.

The SWIM data provided a means by which oxygen saturation (Y) in the veins can be measured from the bulk susceptibility difference (ΔΧ) between tissues [33] (ΔΧ is expressed in parts per billion; ppb). The susceptibility difference ΔΧ of major veins is directly related to the venous blood oxygen saturation (Y) through the following relationship:

$$\Delta Χ=\Delta {Χ}_{do}\times Hct\times \left(1-Y_{v}\right)$$ (1)

where ΔΧ is the susceptibility measurement of major veins, Y_v_ is venous oxygenation, ΔΧ_do_ is the susceptibility difference per unit hematocrit between fully deoxygenated blood and fully oxygenated blood (4π*0.27 ppm [52]), and Hct is the fractional hematocrit in the vein, which is approximately 40% [33].

The relative changes of oxygen saturation in mTBI patients can be calculated using the following simplified equation where all constants have cancelled out:

$$\frac{\Delta Y}{1-Y_{n}}=\frac{\Delta {Χ}_{p}}{{Χ}_{n}}$$ (2)

where ΔY is the oxygenation difference between mTBI patients and normal controls, Δχ_p_ is the susceptibility difference between mTBI patients and normal controls, and χ_n_ is the susceptibility value of normal controls. If the oxygen saturation of veins in the normal healthy controls (Y_n_) is assumed to be 0.7 [53,54], then relative oxygenation change ΔY could be found from this relationship.

**Fig 2:**
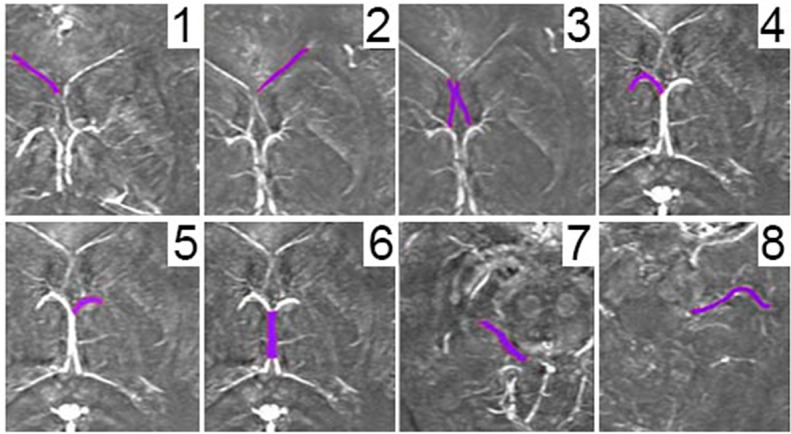
Major veins selected for susceptibility analysis. 1) Left spetal vein; 2) right septal vein; 3) central septal veins; 4) left thalamostriate vein; 5) right thalamostriate vein; 6) internal cerebral vein; 7) left basal vein of Rosenthal; and 8) right basal vein of Rosenthal.

Major veins were selected for analysis (Fig. 2). These included the left, right and central septal veins, the left and right thalamostriate veins, the internal cerebral vein, and the left and right basal veins of Rosenthal. A semi-automated approach was adapted to obtain the relative susceptibility values. Baseline values were selected for each subject by taking the average of 3 measures of means of background brain tissue intensities. ROIs were manually selected for each vessel with a low pass filter cut off of 25% of the baseline value. For each ROI, minimum, maximum, mean, standard deviation and total number of pixels were recorded.

### Neurocognitive testing

At the acute setting, once a patient was conscious and deemed to be clinically capable of participating in the study, neurocognitive testing and a survey with a focus on post-concussion symptoms (PCS) were administered. Given the emergency care setting, it was not feasible to perform a full battery of neuropsychological assessment. Instead, a short instrument called Standardized Assessment of Concussion (SAC) [43] was used to assess the patients’ neurocognitive status. The SAC instrument was originally developed for onsite testing of subject’s neurocognitive performance after sports concussion [55]. SAC is sensitive to the acute changes following concussion and it only requires limited training to administer [56]. The SAC assesses 4 cognitive domains including orientation, attention, immediate memory and delayed recall, and the resulting scores are added to give a patient score between 0 and 30, with a lower score indicating greater cognitive deficit. Previous studies reported SAC to be sensitive to brain injury in the emergency care setting, particularly the delayed recall [57]. The Emergency Room Edition of the SAC instrument also has a graded symptom checklist with all PCS symptoms listed. Patients were asked to grade each symptom from none, mild, moderate, to severe, (rated from 0 to 3, respectively). The points were added to give the overall PCS score.

## Results

### Structural MRI findings

Most patients and controls had no findings on T1 and T2 FLAIR imaging. Two patients and three controls had non-trauma-related findings: arachnoid cyst, pericoallosal lipoma, pineal gland cyst, capillary telangiectasia, and calcification of the falx, respectively. A moderate sized bleed was identified in the gray matter of the right parietal lobe in patient number 4, opposite of scalp edema, and patient 9 showed two small bleeds in the ventrical and cortical parenchyma. Nonspecific hyperintensities were found in 2 patients and 5 controls. These results are summarized in Table 1.

### Susceptibility and blood oxygenation differences between patients and controls

**Fig 3:**
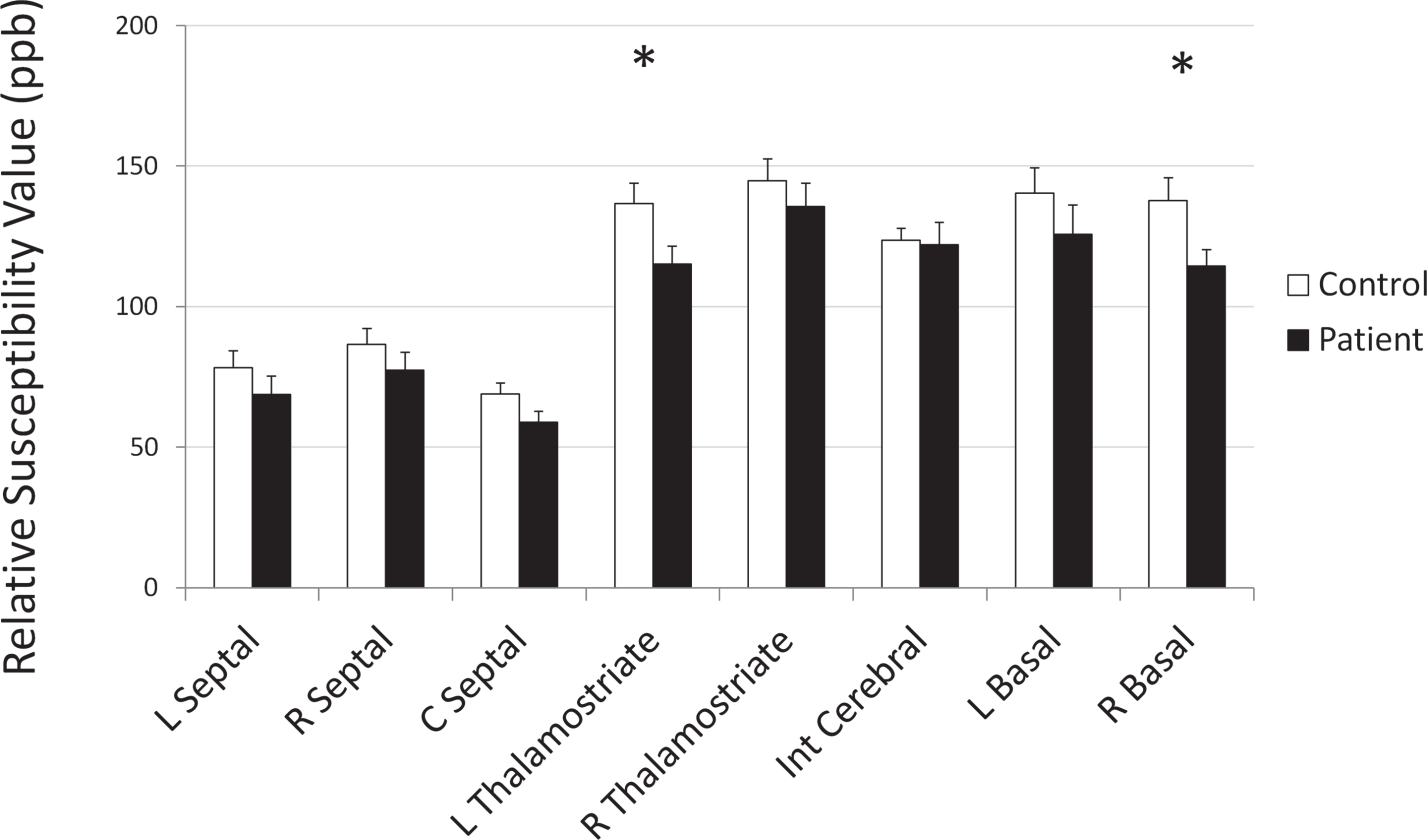
Mean susceptibility values and standard error in major veins. * indicates statistically significant difference between controls and acute visit; † indicates statistically significant difference between acute visit and one-month follow up. R: right, L: left, Int: internal.

A group comparison between the patients with mTBI and the controls was conducted. A two-tailed student’s t-test was performed. The left thalamostriate vein (p = 0.03) and right basal vein of Rosenthal (p = 0.05) showed significantly decreased susceptibility values in patients with mTBI when compared to the control group. Fig. 3 shows the comparison of mean values with standard error bars for mTBI patients and controls. Table 2 shows the group mean values for each vein and student’s t-test comparison between control and patient groups.

**Table 2:** Group mean values for each vein and student’s T-test comparison between control & patient groups and between control and follow-up groups.

	Controls mean (SD)	Patients mean (SD)	T-Test patient vs. control	Patient ΔYp (%)
R Septal	86.58 (23.87)	77.36 (22.31)	0.272	3.19
L Septal	78.33 (25.88)	68.77 (22.71)	0.294	3.66
C Septal	68.85 (17.34)	58.91 (13.26)	0.098	4.33
R Thalamostriate	144.79 (33.10)	135.65 (28.80)	0.423	1.89
L Thalamostriate	136.70 (30.58)	115.06 (22.83)	0.037*	4.75
Int Cerebral	123.58 (18.68)	122.06 (27.77)	0.874	0.37
R Basal	137.78 (34.46)	114.46 (20.58)	0.039*	5.08
L Basal	140.38 (38.50)	125.80 (35.85)	0.279	3.12

* and bold font indicates significant difference. L = left, R = right.

### Inter-rater reliability

Intra-rater and inter-rater correlations were performed for SWIM analysis in 8 randomly chosen patients, to assess the reliability and reproducibility of the method. For inter-rater reliability, a fresh rater was trained on the same process and unaware of the previous results. The correlation (R^2^ value) was 0.98, demonstrating that this study can be repeated with consistency across users. The intra-reader consistency was checked by the same user one week after the first analysis. The intra-reader tests showed correlation of 0.99 indicating excellent reliability of the analysis method.

### ASL differences between patients and controls

**Fig 4:**
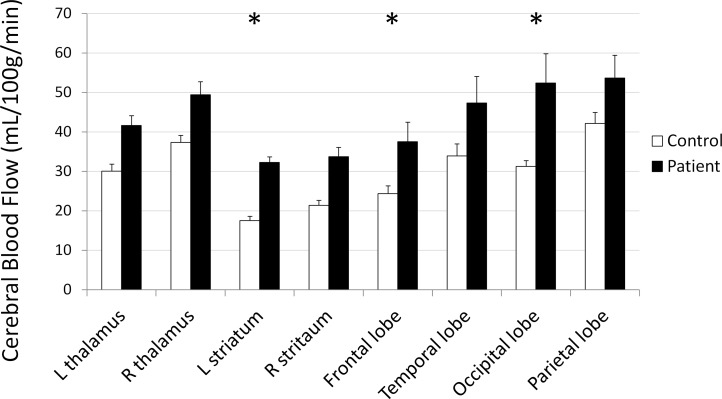
Mean regional rCBF in the thalamus and striatum and cortical lobes of control and patient groups, with standard error bars. * p < 0.05.

We observed significantly higher rCBF values in left striatum of mTBI patients as compared to controls (*p* = 0.01, student t test). In particular, the caudate, putamen, and pallidum rCBF were significantly increased. We also observed elevated rCBF values in left frontal and occipital lobes (*p* = 0.03 for both, student t test; Table 3, Fig. 4). No significant changes in the thalamus, globus pallidus, or temporal or parietal lobes were found (all *p*>0.05 for student t-tests).

**Table 3:** Comparison of mean CBF values (in mL/100g/min) between control and patient groups.

Region	Controls mean (SD)	Patients mean (SD)	p value
Left thalamus	30.03 (6.25)	41.61 (6.71)	0.22
Right thalamus	37.33 (6.17)	49.44 (8.77)	0.28
Left striatum	17.49 (4.01)	32.27 (3.72)	0.01*
Right striatum	21.4 (4.31)	33.69 (6.38)	0.14
Frontal lobe	24.38 (6.83)	37.54 (13.09)	0.03*
Temporal lobe	33.88 (10.81)	47.31 (17.97)	0.11
Occipital lobe	31.27 (5.02)	52.42 (19.63)	0.03*
Parietal lobe	42.14 (9.8)	53.66 (15.33)	0.10

p value shows level of significance for student’s t-test.

* and bold font indicates significant difference.

### Neurocognitive testing

**Fig 5:**
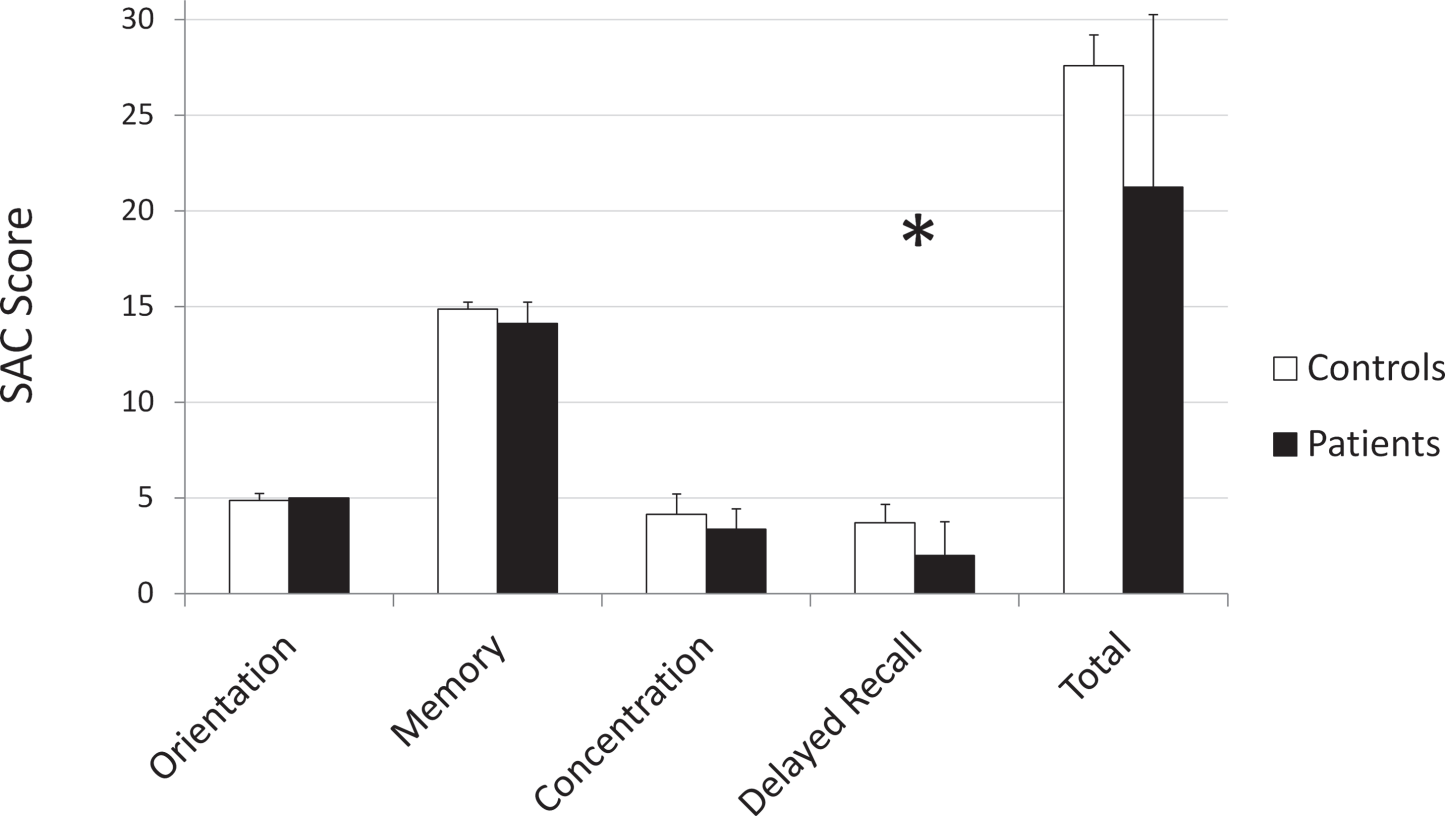
Group differences in SAC scores between controls and mTBI patients. * p < 0.05.

Given the small sample size, mTBI patients’ SAC scores were compared with a normalized dataset reported by McCrea et al., of 568 healthy controls [43]. mTBI patients' delayed recall scores were significantly lower than the normalized dataset on the delayed recall score (p = 0.04; Table 4, Fig. 5). Other measures were not significantly different from controls.

**Table 4:** mTBI patients' SAC scores compared with normalized SAC scores.

	Orientation mean (SD)	Memory mean (SD)	Concentration mean (SD)	Delayed Recall mean (SD)	Total Score mean (SD)
Controls (N = 568)	4.82 (0.43)	14.51 (0.98)	3.40 (1.27)	3.84 (1.11)	26.58 (2.23)
Patients (N = 7)	5 (0)	14 (1.15)	3.29 (1.11)	2 (1.91)	24.28 (2.98)
2-tailed T-Test (*p* value)	0.00	0.28	0.8	0.04*	0.08

* and bold font indicates significant difference.

No significant correlations were found between SAC/Post Concussion Syndrome (PCS) scores and the rCBF values of all of the above mentioned regions.

### Potential confounders

Patients’ blood alcohol levels were tested at the discretion of the attending emergency physicians in any case of suspected alcohol intoxication. Of the 14 patients in this study, one patient out of five tested had detectable alcohol in the blood in the ED, at 46 mg/dl. Eight patients received opiates as part of their the ED treatment, four of whom had tested positive for opiates or benzodiazepines and two of whom tested positive for cannabinoids, and one patient received benzodiazepines. No other patients tested positive for opiates or benzodiazepines. Three patients received intravenous (IV) normal saline, and another received IV potassium chloride. Three reported a diagnosis of hypertension, while five had a systolic BP over 140 in the ED, including two patients with a systolic BP over 160.

Because of the small sample size, there was exceedingly low power for detecting effects of any of these as confounders, but there were no significant differences in susceptibility between patients with mTBI who did (n = 8) or did not receive (n = 6) opiates in ER (left thalamostriate vein p = 0.84, right basal vein of Rosenthal p = 0.70), between subjects who did (n = 3) or did not (n = 11) receive normal saline (left thalamostriate vein p = 0.46, right basal vein of Rosenthal p = 0.07), or between subjects who did (n = 4) or did not have (n = 10) positive opiate or benzodiazepine drug screen (left thalamostriate vein p = 0.24, right basal vein of Rosenthal p = 0.67). For the patients who received ASL imaging, there were no significant differences in rCBF between subjects who did (n = 2) or did not receive (n = 5) opiates in ER (left striatum p = 0.60, frontal lobe p = 0.73, and occipital lobe p = 0.96) or between subjects who did (n = 2) or did not receive (n = 5) normal saline (left striatum p = 0.29, frontal lobe p = 0.61, and occipital lobe p = 0.45). In summary, none of these possible confounders were found to have a significant effect on measured rCBF or susceptibility.

## Discussion

To the best of our knowledge, this is the first study reporting cerebral hemodynamic changes using a non-invasive MRI method in the acute stage of mTBI. Our have demonstrated that, as a result of concussion, the brain has increased rCBF and consequently higher venous oxygenation. This finding is in opposition to our original hypothesis that the brain may have decreased rCBF and decreased venous oxygenation. This is a novel finding in patients with mTBI in the acute post-TBI stage.

Results of ASL data analysis indicate that the left striatum has significantly higher rCBF in the patient than in the control group. This is in contrast to several studies indicating decreased rCBF after moderate-severe TBI [57–60]. This is likely the result of the differences between these studies and ours in both severity of the injury and timing of the scanning. All of our patients were classified as having mTBI and most were scanned at the acute stage (within 48 hours after injury), which may be expected to show different pathophysiology than the subacute or chronic stages of moderate or severe TBI. To our knowledge, such ultra-early scanning in this population has not been attempted in the past.

Although the increase in rCBF contradicts some of the earlier publications, there are a few studies indicating higher CBF after trauma [61–67]. Marion et al., observed that CBF peaked to a significant high at the 24 hour time point after injury [61], but studied a diverse range of injury pathologies at a wide range of times after injury. Obrist et al., studied the cerebral metabolic rate of oxygen (CMRO_2_) and CBF in 75 severely-injured patients and observed a positive correlation between GCS score and CBF in patients with far lower GCS scores than ours. They also found that CBF reached its highest peak at 24 hours after injury [68], supporting the same timeline as seen in Marion's study. Muizelaar et al., suggested that CMRO_2_ is significantly positively correlated with GCS scores [64], though the study was limited to severe cases and therefore did not include patients with GCS scores as high as our patients had. Bouma et al., also observed that relative CBF reaches its peak between 24 to 48 hours after injury [62,65], which was supported by another study by the same group [63] and also by the results of the study by Mendelow et al., [69]. In an animal study, Prat et al., observed that the animals impacted with less weight (producing a less severe injury) showed an increase in CBF between one and three hours after injury, which was followed by a decrease in CBF thereafter [60].

SWI analysis indicates that relative susceptibility values in the veins of patients are lower than in the control group. In particular, the left thalamostriate vein and the right basal vein of Rosenthal had significantly lower susceptibility. This implies that there is more oxygen left in the veins in patients as compared to the control group, in contrast to earlier animal studies [70]. A study by Shen et al., found increased susceptibility induced by head injury using the Marmarou rat model [71]. However, the severity and mechanism of the injury in Shen's study differs from ours. Considering this, there could be several explanations for the decreased relative susceptibility. One is that the decreased susceptibility could represent a deficit in oxygen consumption at tissue level after injury. In several studies it has been shown that the coupling between CMRO_2_ and CBF has been disrupted following injury [72–75]. Decreased CMRO_2_ has been observed in these studies, suggesting that either the tissue is unable to absorb the optimal amount of oxygen or that there is a reduction in oxygen demand. Another proposition that might explain the lower relative susceptibility value is that there might be increased rCBF in the regions drained by those veins; an increase in rCBF with stable CMRO_2_ would result in decreased oxygen extraction and more oxygen left in the veins.

A fourth option is that the brain is utilizing another source of energy, such as lactate, instead of glucose. Several studies have shown this to occur in extreme conditions such as starvation and trauma [76], using substrates such as b-hydroxybutyrate (bHB) [77] or lactate [78]. A possible mechanism behind this could be efforts by the brain to maintain energy production in the presence of mitochondrial dysfunction and decreased CMRO_2_. There have been few studies showing decreased CMRO_2_ after TBI [79]. In the absence of any structural damage visible on CT or MRI, this could represent a temporary inability of the brain to use oxygen efficiently via glucose uptake. It is possible that the brain is attempting to overcome mitochondrial dysfunction and lowered CMRO_2_ by increasing CBF.

It is also possible that this increase in CBF is a neuroprotective mechanism that is hindered by or lost in more severe injuries in which CBF is found to be decreased. Mild mitochondrial dysfunction leads to an increase in anaerobic ATP production in order to meet demands. The brain attempts to maintain the energy equilibrium in the acute stage, which requires an increase in CBF to offset a decrease in efficiency of ATP production in affected areas of the brain. This decoupling of CBF and CMRO_2_ explains the combination of increased CBF and increased venous oxygenation.

Unfortunately, the strong similarity between patient and control group SAC scores led to relatively low sensitivity to detect a correlation between rCBF or SWIM abnormalities and symptom presence or severity. Despite the advantage in ease of administering the SAC questionnaire, a more sensitive questionnaire or neuropsychological test should be applied in the future to more accurately characterize the deficits experienced by patients with mTBI.

There are several approaches to extracting oxygen saturation, including T2, T2* or T2' approaches [15–31,33,80–86], phase-based methods [32–37,52], and susceptometry [49]. All these methods have advantages and disadvantages. All of these methods are interesting, but they all meet with difficulties, such as with phase wrapping. Additionally, none of these are in common clinical use today except for susceptibility weighted imaging (SWI) and the phase approach leading to SWIM. By evaluating each major vein inside the brain parenchyma, we will be able to determine the blood oxygenation at a regional level. Similar to a brain catheter probe measuring surrounding brain tissue oxygenation, SWIM uses the major veins of the brain like embedded catheter probes for detecting surrounding brain tissue oxygenation, as well.

Veins vary in size from roughly 1 cm in diameter for the dural sinuses, to a few millimeters for pial veins, to only several hundred microns for medullary veins [87]. Our susceptibility measurements of major veins (between 70 and 150ppm) are significantly lower than that in superior sagittal sinus (SSS) measured in susceptometry approach. The different susceptibilities in veins with different sizes are due to its partial volume effects [88]. In looking at the relative changes of venous oxygenation between patients and controls, this partial volume effect may downplay the group difference instead of exaggerating the confounding factors. In comparison, a susceptometry approach using the SSS does not suffer partial volume effect but it only offers global oxygenation instead of regional information. Furthermore, since the SSS is located at the interface of brain tissue and skull, the strong artifact on phase images makes the SSS not ideal to evaluate on the current 3D SWI data. Additionally, the brain extraction step during SWIM processing often removes a small portion of the outer cortex along with the skull, as this area is generally noisy in the phase images. Part of the SSS is usually removed along with this, while the deeper veins of the brain are unaffected. For these reasons, we chose to analyze the deeper veins instead of the SSS; while they offer slightly lower SNR, we have greater confidence in the accuracy of the values obtained.

Our current study has several limitations that need to be discussed. First, our relatively small sample size provides low power. With a larger sample size, some of the relationships that are present in the data may turn out to be not significant. We are currently in the process of expanding this data set, and intend to investigate these relationships further. In addition, while the recruitment of the first few patients had a wide range delays between injury and MRI (with one subject having their first scan 10 days after injury, potentially confounding the results), subjects recruited later had their MRI within a much shorter time window, which should reduce some of the variability. The initial patients are still being followed up, as well, which will allow for a more longitudinal analysis of their recovery. Additionally, we will acquire more detailed neuropsychological data in our control group for a more relevant comparison. We also acknowledge that the SAC is considered as a sideline test to supplement a full battery of neuropsychological test. In a typical acute setting, it is not possible to hold the patient for an hour-long full battery neuropsychological test, which is also a confounding factor of the study. Finally, we must consider that the increase in rCBF could be a result of some other difference between the patients with mTBI and the control group like, for example, the stress related to injury present in patients with mTBI but not healthy controls enrolled in the study. Further, because of the difficulties in recruiting orthopedically injured controls from the ED, as well as increased difficulties with follow up as compared to control subjects recruited from the surrounding community, the patients may have received interventions which affect CBF that the controls have not. While none of the possible confounders identified were found to have a significant effect in this dataset, this possibility should be considered in a larger dataset that would provide more power for detecting or disproving the presence of such differences. One notable possible confounder is IV administration of normal saline, commonly given to most patients in the ED. Some studies have shown this to have an effect on CBF [89,90], but these studies have been performed in humans and animals with far different conditions than the patients in this study, including subarachnoid hemorrhage and cardiac arrest. What we are currently lacking are studies of the effects of normal saline on rCBF in normovolemic, non-hypoxic patients with normal intracranial pressure. We do not anticipate that standard, IV-administered saline will have a significant effect on CBF, which is supported by the changes in CMRO2; if the CBF changes were artifacts from volume expansion or increased cardiac output, we would not expect the CMRO2 to change.

In the future, we plan to take these analyses a step further and to calculate the cerebral metabolic rate of oxygen (CMRO_2_) for all patients scanned. This will require the addition of several 2D SWI sequences with higher resolution, in order to obtain absolute quantification while avoiding partial volume effects in very small veins. This measurement will allow us to better describe the metabolic status of the tissue.

In summary, imaging of changes in hemodynamics and metabolism following mTBI can clearly provide us with more information about the pathophysiology that underlies the symptoms, progression, and recovery of brain injury. In this study, we demonstrated that patients with mTBI have increased cerebral blood supply as a potential compensatory mechanism to cope with brain injury at the acute stage. Further investigation in larger cohort is needed to establish the relationship between rCBF and tissue oxygenation and its neurocognitive underpinnings.
