# Correction: Cerebral Hemodynamic Changes of Mild Traumatic Brain Injury at the Acute Stage

**DOI:** 10.1371/journal.pone.0127487

**Published:** 2015-05-01

**Authors:** The PLOS ONE Staff

There are errors in the title for Table 1. The correct title is: Patients’ and controls’ demographic data and cause of injury. Please see the corrected Table 1 here.

**Table 1:** Patients’ and controls’ demographic data and cause of injury.

Case ID	Age (Years)	Sex	Race	Delay to Scan	ER GCS	Injury Mech.	ASL	SWI	Structural findings
Patients									
001	27	F	Caucasian	41 hr		MVA	X	X	
2	29	M	Caucasian	10 d	15	MVA		X	
002	24	M	Caucasian	46 hr		Fall		X	
4	20	M	Caucasian	27 hr	15	Fall		X	Moderate bleed, scalp edema
6	21	M	Black	4 d		MVA		X	
7	25	M	Black	17 hr	15	Assault	X	X	Nonsp hyperint, scalp edema
9	56	M	Indian	26 hr	15	MVA		X	Nonsp hyperint, 2 small bleeds
11	35	M	Black	36 hr		Fall		X	
14	30	M	Caucasian	7 d	15	Fall	X	X	
15	36	F	Black	9 hr	15	MVA	X	X	Arachnoid cyst
16	19	M	Black	3 hr	15	MVA	X	X	Pericallosal lipoma
17	23	M	Black	9 hr	15	MVA	X	X	
18	21	F	Caucasian	2 d	15	MVA		X	
19	30	F	Asian	8 hr	15	SBV	X	X	
Mean	27.14			55.29					
SD	5.52			68.82					
Median	26			31.5 hr					
Range	19–56			3 hr–10 d					
Controls									
1	24	F	Asian				X	X	
2	23	M	Indian				X		
3	45	M	Caucasian					X	Nonsp hyperint
5	22	M	Caucasian					X	Capillary telangiectasia
6	27	M	Asian				X	X	
7	23	F	Asian				X	X	
8	22	F	Asian				X	X	
9	65	F	Asian					X	Pineal gland cyst, Nonsp hyperint
36	52	F	Caucasian				X	X	Nonsp hyperint
37	44	M	Caucasian				X	X	Calcification of falx
38	41	M	Caucasian				X	X	
39	28	F	Caucasian					X	
40	27	F	Arabic				X	X	
41	29	M	Caucasian				X	X	
42	33	M	Caucasian				X	X	
43	66	F	Caucasian					X	Nonsp hyperint
44	38	M	Hispanic					X	Nonsp hyperint
45	28	M	Caucasian					X	
46	21	M	Black				X	X	
Mean	30.08								
SD	10.24								
Median	28								
Range	21–66								

There is an error in the legend for Fig 3. Please see Fig 3 and its complete, correct legend here.

**Fig 3:**
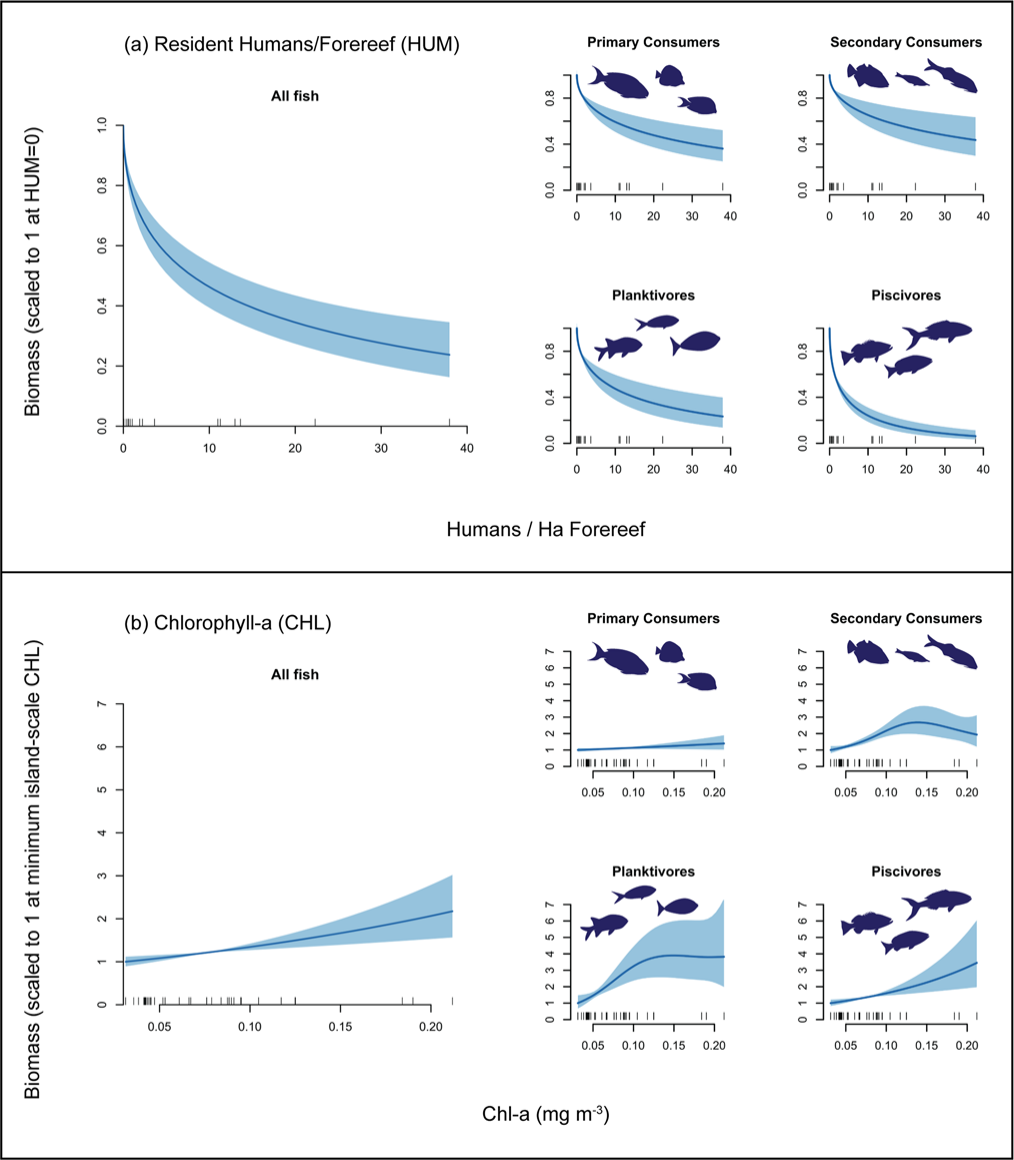
Mean susceptibility values and standard error in major veins. *indicates statistically significant difference between controls and patients.

There are errors in the title and the key for Table 2. Please see the corrected Table 2 here.

**Table 2:** Group mean values for each vein and student’s T-test comparison between control and patient groups.

	Controls	Patients	T-Test patient vs.	Patient ΔYp
	mean (SD)	mean (SD)	control	(%)
R Septal	86.58 (23.87)	77.36 (22.31)	0.272	3.19
L Septal	78.33 (25.88)	68.77 (22.71)	0.294	3.66
C Septal	68.85 (17.34)	58.91 (13.26)	0.098	4.33
R Thalamostriate	144.79 (33.10)	135.65 (28.80)	0.423	1.89
L Thalamostriate	136.70 (30.58)	115.06 (22.83)	0.037*	4.75
Int Cerebral	123.58 (18.68)	122.06 (27.77)	0.874	0.37
R Basal	137.78 (34.46)	114.46 (20.58)	0.039*	5.08
L Basal	140.38 (38.50)	125.80 (35.85)	0.279	3.12

* indicates significant difference. L = left, R = right.

There are errors in the key for Table 3. Please see the corrected Table 3 here.

**Table 3:** Comparison of mean CBF values (in mL/100g/min) between control and patient groups.

	Controls	Patients	
Region	mean (SD)	mean (SD)	p value
Left thalamus	30.03 (6.25)	41.61 (6.71)	0.22
Right thalamus	37.33 (6.17)	49.44 (8.77)	0.28
Left striatum	17.49 (4.01)	32.27 (3.72)	0.01*
Right striatum	21.4 (4.31)	33.69 (6.38)	0.14
Frontal lobe	24.38 (6.83)	37.54 (13.09)	0.03*
Temporal lobe	33.88 (10.81)	47.31 (17.97)	0.11
Occipital lobe	31.27 (5.02)	52.42 (19.63)	0.03*
Parietal lobe	42.14 (9.8)	53.66 (15.33)	0.10

p value shows level of significance for student’s t-test.

* indicates significant difference.

There are errors in the key for Table 4. Please see the corrected Table 4 here.

**Table 4:** mTBI patients' SAC scores compared with normalized SAC scores.

	Orientation mean (SD)	Memory mean (SD)	Concentration mean (SD)	Delayed Recall mean (SD)	Total Score mean (SD)
Controls (N = 568)	4.82 (0.43)	14.51 (0.98)	3.40 (1.27)	3.84 (1.11)	26.58 (2.23)
Patients (N = 7)	5 (0)	14 (1.15)	3.29 (1.11)	2 (1.91)	24.28 (2.98)
2-tailed T-Test (*p* value)	0.00	0.28	0.8	0.04*	0.08

* indicates significant difference.

There is an error in the second sentence of the Discussion. The correct sentence is: We have demonstrated that, as a result of concussion, the brain has increased rCBF and consequently higher venous oxygenation.
