# Correction: Correction: Cerebral Hemodynamic Changes of Mild Traumatic Brain Injury at the Acute Stage

**DOI:** 10.1371/journal.pone.0132750

**Published:** 2015-07-13

**Authors:** The PLOS ONE Staff

The image for Fig 3, “Mean susceptibility values and standard error in major veins”, is incorrect in the correction published on May 1, 2015. The publisher apologizes for this error. Please view Fig 3 here.

**Fig 3:**
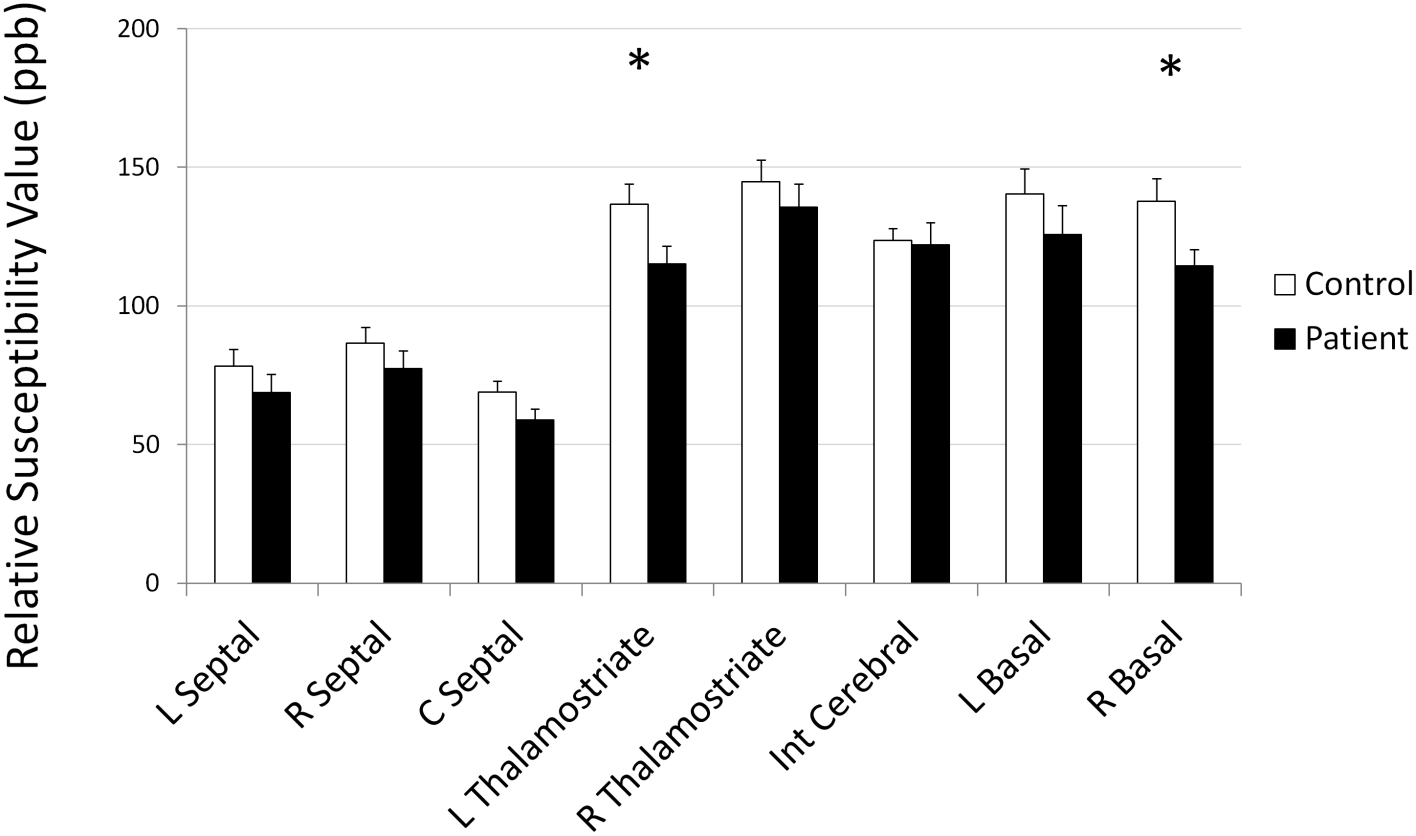
Mean susceptibility values and standard error in major veins. *indicates statistically significant difference between controls and patients.
